# Caregiver burden and familial impact in Down Syndrome Regression Disorder

**DOI:** 10.1186/s13023-025-03644-0

**Published:** 2025-03-14

**Authors:** Katherine Chow, Panteha Hayati Rezvan, Lilia Kazerooni, Lina Nguyen, Natalie K. Boyd, Benjamin N. Vogel, Maeve C Lucas, Ruth Brown, Eileen A. Quinn, Saba Jafarpour, Jonathan D. Santoro

**Affiliations:** 1 Keck School of Medicine of the University of Southern California, Los Angeles, CA USA; 2 Biostatistics and Data Management Core, The Saban Research Institute, Children’s Hospital Los Angeles, Los Angeles, CA USA; 3 Division of Neurology, Department of Pediatrics, Children’s Hospital Los Angeles, Los Angeles, CA USA; 4 Virginia Institute for Psychiatric and Behavioral Genetics at Virginia Commonwealth University, Richmond, VA USA; 5 Division of Developmental and Behavioral Pediatrics, University of Toledo College of Medicine and Life Sciences, Toledo, OH USA; 6 Department of Neurology, Keck School of Medicine of the University of Southern California, Los Angeles, CA USA; 7 Department of Pediatrics, Children’s Hospital Los Angeles, 4650 Sunset Blvd, Mailstop 82, Los Angeles, CA 90027 USA

**Keywords:** Down syndrome, Regression, Caregiver, Quality of life, Depression, Neurologic, Burden

## Abstract

**Background:**

Down Syndrome Regression Disorder (DSRD) is an acute or subacute neurocognitive regression in individuals with Down syndrome (DS), characterized by a loss of previously acquired cognitive, adaptive, and social skills. DSRD profoundly affects individuals’ ability to engage in activities of daily living, making them highly dependent on their caregivers who must provide significantly more support than before the DSRD diagnosis. This study aimed to examine caregiver burden, quality of life, and depression among caregivers of individuals with DSRD versus caregivers of those with DS and other neurological disorders (DSN).

**Design/Methods:**

In this cross-sectional study, caregivers of individuals with DSRD (*n* = 228) and DSN (*n* = 137) were recruited through Children’s Hospital Los Angeles neurology clinic and a Facebook DSRD support group. Participants completed standardized questionnaires assessing quality of life (PedsQL Family Impact Module), caregiver burden (Zarit Caregiver Burden Assessment, ZCB), and depression (Glasgow Depression Scale, GDS), along with additional items addressing other factors of caregiver well-being. Data were analyzed using bivariate comparisons and univariate regression models to assess differences between groups.

**Results:**

Caregivers of individuals with DSRD were more likely than caregivers of those with DSN to report increased financial burden (*p* = 0.003), housing changes (*p* = 0.02), disrupted sleep (*p* < 0.001), negative impacts on social networks (*p* < 0.001), and worsened mental health (*p* < 0.001). Furthermore, DSRD caregivers reported significantly higher levels of burden (mean difference [95% CI]: 8.3 [6.3, 9.7]) and depression symptoms (2 [0.7, 3.4]), reflecting greater perceived stress and burden. They also had lower quality of life scores (-27.9 [-30.2, -25.5]), indicating a more substantial impact on overall well-being and daily functioning compared to DSN caregivers. Additionally, caregivers in the DSRD group had higher odds (odds ratio [95% CI]: 4.7 [2.9, 7.7)] of meeting clinical depression criteria (GDS score ≥ 13) than caregivers in the DSN group.

**Conclusions:**

Caregivers of individuals with DSRD experience significantly greater distress and burden compared to those caring for individuals with DSN. The elevated risk of depression, combined with reduced quality of life and increased burden, underscores the multimodal nature of the challenges faced by this population.

**Supplementary Information:**

The online version contains supplementary material available at 10.1186/s13023-025-03644-0.

## Background

Down Syndrome Regression Disorder (DSRD) is an acute or subacute neurocognitive regression in individuals with Down syndrome (DS), characterized by a loss of previously acquired cognitive, adaptive, and social skills in the 2nd or 3rd decade of life [1, 2]. Symptoms include regression or loss of language, communication, cognition, and executive function, and can also include psychiatric manifestations, bradykinesia, catatonia, and rapid-onset insomnia [1, 2]. The severe decline in functioning caused by DSRD can be devastating, not only impairing the quality of life and autonomy of individuals with DS, but also significantly affecting their caregivers and community.

DSRD results in a profound loss of ability to engage in activities of daily living, to the point where individuals often become dependent on their caregivers. This can cause caregivers to experience sleep disturbances, financial distress, and negative impacts to family dynamics and social networks, among other challenges [3–8]. Providing care is incredibly time intensive and individuals with DSRD require considerably more support than they did before their diagnosis. In individuals with DS, multiple studies have reported an increased caregiver burden when compared to caregivers of neurotypical children [9]. Individuals with DS have developmental delays and multiple chronic medical conditions, which require increased caregiver attention and care [10, 11]. Individuals with DS rely heavily on their caregivers for many of their activities of daily living, posing significant responsibility to caregivers [9]. Since DSRD has additional unique aspects that include the sudden and severe loss of skills, DSRD may have distinct challenges that further intensify caregiver distress.

The purpose of this study is to elucidate the multifaceted impacts of DSRD on caregivers and families. Specifically, it aims to: (1) compare individuals with DSRD to those with DS and other neurological disorders (DSN) regarding demographic and clinical characteristics, while assessing differences in quality of life, depression, and burden among their families and caregivers; and (2) investigate potential factors that may contribute to these possible differences. Understanding the extent and nature of familial distress in this context is crucial for developing targeted interventions and support systems to alleviate caregiver burden and enhance family resilience.

## Methods

### Regulatory approval and data availability

This study was approved by the institutional review board at Children’s Hospital Los Angeles and the University of Southern California (IRB number: CHLA-24-00184). Virtual consent was obtained by caregivers or guardians. This consent consisted of a standardized webpage detailing the nature of the study, anticipated duration, and risks/benefits of participation with options to participate or not for all individuals accessing the survey. Anonymized data is available upon request to qualified researchers pending IRB approval.

### Participants

There were two unique cohorts in this study sample: (1) DSRD Cohort: Caregivers of individuals with DSRD were recruited from CHLA neurology clinic and via virtual recruitment on a Facebook support group for individuals and caregivers of persons with DSRD. The recruitment period lasted 3 weeks (July 6, 2024, to July 28, 2024). Participants were eligible if they had a child or family member who had a diagnosis of possible or probable DSRD. (2) DSN Cohort: Caregivers of individuals with a diagnosis of DS and any of the following neurologic conditions (i.e., moyamoya vasculopathy, epilepsy, and autism spectrum disorder (ASD)) were recruited during the same study period. Patients had to have an active neurologic problem that was symptomatic at the time of the survey being completed. To be eligible for this study, caregivers in both cohorts had to be over 18 years old and generally knowledgeable about their loved one’s condition.

### Confirmation of diagnosis

All caregiver responses for individuals with DSRD were evaluated post-hoc to confirm that the caregiver report of symptoms was consistent with consensus criteria for the diagnosis of DSRD [2]. The survey included the DSRD criteria [2] verbatim which was organized by the presence or absence of sypmtoms in large categories (e.g., movement disorder) and respondents were asked to report if these criteria were met at the onset of symptoms. This approach aimed to minimize the possibility of collecting data on individuals without DSRD in that cohort and ensure accurate identification of individuals meeting the criteria. This was performed by an independent medical reviewer, who only reviewed clinical data and was not privy to demographic data. Caregivers of individuals who did not meet the minimum of three core symptoms (minimum threshold for possible DSRD diagnosis) had their responses excluded. As the survey was anonymous, confirmation of clinical symptoms or further interrogation regarding symptoms was not possible.

In the DSN cohort, diagnosis of the neurological condition was by self-report only. Individuals in this cohort required a physician-based diagnosis in all cases.

### Survey

Caregivers completed an anonymous, voluntary online survey via REDCap on their levels of distress and burden due to the DSRD diagnosis [12, 13] (Appendix A). The first page of the survey was a consent with viewing of the questionnaire only available after completion of consent. Demographic and clinical data were collected for all participants as it related to the individual with DSRD or DSN. Multiple objective surveys were administered to assess caregiver quality of life (Pediatric Quality of Life [PedsQL] Family Impact Module), depression (Glasgow Depression Scale, GDS), and caregiver burden (Zarit Caregiver Burden Assessment, ZCB) [14–16].

#### PedsQL family impact module version 2.0

This standardized and well-validated tool assesses the impact of pediatric health conditions on parents and families and has been validated in various populations [16]. This assessment is widely used to evaluate caregiver burden and family functioning, providing insights into the emotional, social, and physical toll of caregiving. The PedsQL FIM is a 36-item questionnaire with 8 different domains: physical functioning (6 items), emotional functioning (5 items), social functioning (4 items), cognitive functioning (5 items), communication (3 items), worry (5 items), and daily activities (3 items). The PedsQL utilized a 5-point Likert scale with options 0 (never a problem) to 4 (almost always a problem). Total scores are calculated by reverse scoring the items and linearly transforming them to a 0-100 scale. Total scores are calculated by summing all 36 items and dividing by the total number of items answered. Domain scores are obtained by averaging the scores within each domain, providing insight into specific areas. The parent HRQL (Health-Related Quality of Life) summary score is computed as an average of 20 items from the physical, emotional, social, and cognitive functioning scales, while the family functioning summary score is calculated as the mean of 8 items from daily activities and family relationships scales. All scores are based on items answered, with higher scores indicating better adaptive functioning [16].

#### GDS

The GDS was an optional additional mental health questionnaire that was used to evaluate the presence and severity of depressive symptoms in caregivers. The GDS consists of 20 items that ask about experiences over the past week, with response options of 0 (never/no), 1 (sometimes), and 2 (always/a lot). The total score is calculated by summing the scores across all items. Higher scores indicate more severe depressive symptoms, with a score of 13 or higher indicating clinically significant depression. The GDS was dichotomized into two categories (≥ 13 vs. < 13) to differentiate caregivers with clinically significant depression from those without [14].

#### ZCB

The ZCB is a widely used and well-validated questionnaire that was used to assess the level of physical, emotional, and financial burden on caregivers. The ZCB is composed of 22 items which are rated on a 5-point Likert scale with response options from 0 (never) to 4 (nearly always). The total ZCB score is calculated by summing the scores of all 22 items, with total possible scores ranging from 0 to 88. Higher scores indicate a greater level of caregiver burden, with a score over 61 indicating high burden [15].

In addition to the above standardized metrics, a battery of additional questions, the DSRD Caregiver Distress Survey (CDS), was included to capture additional factors affecting caregiver well-being that were not addressed by the established instruments. The CDS consists of 28 questions organized into 6 sections: financial impact (4 items), housing (4 items), sleep (2–4 items), siblings (1–5 items), social networks and relationships (6 items), and mental health (8 items). The survey included questions with a range of response formats, including yes/no, positive/negative/no change, multiple-response (e.g., “check all that apply”), and open-ended responses to gather qualitative insights. Descriptive statistics were collected, and raw scores were converted to a total percent response. Two questions within the social networks and relationships section assessed caregivers’ primary positive support networks before and after loved ones’ DSRD diagnosis, aiming to capture changes in sources of support from 10 options (e.g., friends, family, siblings, etc.). Total scores for positive support networks were calculated by summing selected responses pre- and post-diagnosis, with scores ranging from 0 to 10. Individuals had the option to skip any question in the survey.

The survey automatically closed out for caregivers of individuals with DSRD and DSN after they completed the last metric. Medical records were not accessed for this survey; instead, caregiver-reported demographics, clinical information (e.g., neurological diagnosis in the DSN cohort and symptoms of DSRD in the DSRD cohort [2]), and objective caregiver burden metrics were used. Post-hoc, IP addresses were referenced to ensure that multiple completions of the survey were not performed. Cookies were not used to track responses. Completion rate was determined by dividing the number of surveys completed by the total number of consents obtained.

### Statistical analysis

We initially conducted bivariate analyses to examine differences in demographic and clinical characteristics between individuals with DSRD and DSN, as well as differences in quality of life, depression, and caregiver burden among their families. Chi-square or Fisher’s exact tests were employed for categorical variables, while *t*-tests (or Wilcoxon rank-sum tests for skewed distributions) were used for continuous variables. A repeated measures analysis was conducted to examine changes in total positive support network scores from pre- to post-diagnosis between the DSRD and DSN groups. Univariate regression models assessed associations between disease diagnosis (DSRD vs. DSN) and quality of life, familial distress, and caregiver burden. In particular, univariate linear regression analyses were performed for PedsQL and ZCB scores, logistic regression analysis for GDS (≥ 13 vs. < 13), and quantile regression analysis for GDS total score. We further examined whether potential factors, including increased financial burden, housing changes, disrupted sleep, negative impacts on social networks, and worsened mental health, modified these associations by incorporating interaction terms between disease diagnosis and each factor in the models. Statistical significance was established a priori at 2-sided *p* < 0.05. All analyses were performed in Stata/MP release 18.0 [17].

## Results

### Cohort demographics

In total, 365 individuals completed the survey in full. Of the total participants, 62.5% (*n* = 228) had DSRD, and 37.5% (*n* = 137) had DSN. Caregiver-reported clinical and demographic data regarding the cohorts is presented in Table 1. In the DSRD group, more than two-thirds presented altered mental status (71.1%), movement disorder (70.2%), language deficits (69.7%), psychiatric symptoms (67.1%), and insomnia (65.8%), with nearly half experiencing cognitive decline (49.1%) and only 4.8% having focal neurologic deficits/seizure. On average, individuals with DSRD reported five core “diagnostic” symptoms of the condition (SD = 1.5).^12^

**Table 1:** Demographic and clinical characteristics by DSRD and DSN groups

	DSRD	DSN	Total	*p*
	(*n* = 228)	(*n* = 137)	(*n* = 365)	
*Demographics*				
Age (years)^*^	20.13 (7.38)	16.05 (5.25)	18.60 (6.94)	**< 0.001**
Sex at birth				
Female	111 (48.7%)	60 (43.8%)	171 (46.8%)	0.365
Male	117 (51.3%)	77 (56.2%)	194 (53.2%)	
Ethnicity †				
Non-Hispanic	168 (73.7%)	95 (69.3%)	263 (72.1%)	0.371
Hispanic	60 (26.3%)	42 (30.7%)	102 (27.9%)	
Race †				
White	170 (74.6%)	100 (73.0%)	270 (74.0%)	0.782
Black/African American	32 (14.0%)	19 (13.9%)	51 (14.0%)	
Asian	17 (7.5%)	13 (9.5%)	30 (8.2%)	
Native American/Alaskan Native	7 (3.1%)	5 (3.6%)	12 (3.3%)	
Hawaiian or Pacific Islander	2 (0.9%)	0 (0.0%)	2 (0.5%)	
Duration of symptoms (years)	4.37 (3.80)	4.59 (2.19)	4.45 (3.29)	0.540
*PedsQL*				
Physical functioning score^*^	41.26 (19.56)	70.65 (8.41)	52.29 (21.63)	**< 0.001**
Emotional functioning score^*^	31.43 (18.26)	67.19 (9.94)	44.85 (23.36)	**< 0.001**
Social functioning score^*^	28.43 (21.06)	61.45 (9.24)	40.82 (23.77)	**< 0.001**
Cognitive functioning score ^*^	46.49 (22.48)	75.40 (10.50)	57.34 (23.51)	**< 0.001**
Communication score ^*^	31.80 (20.47)	60.04 (10.26)	42.40 (22.10)	**< 0.001**
Worry score ^*^	20.00 (17.36)	46.75 (9.21)	30.04 (19.70)	**< 0.001**
Daily activities score ^*^	24.42 (19.33)	58.64 (10.57)	37.26 (23.45)	**< 0.001**
Family relationships score ^*^	45.09 (24.81)	55.18 (7.98)	48.88 (20.77)	**< 0.001**
Total score^*^	34.58 (13.68)	62.45 (3.90)	45.04 (17.47)	**< 0.001**
The parent HRQL summary score^*^	37.54 (15.34)	69.13 (5.44)	49.40 (19.81)	**< 0.001**
The family functioning summary score^*^	37.34 (19.00)	56.48 (5.89)	44.52 (18.01)	**< 0.001**
*Zarit* caregiver burden total score^*^	30.92 (7.92)	22.62 (2.53)	27.80 (7.59)	**< 0.001**
*Glasgow* depression score^*^	13 [0, 17]	11 [10, 12]	12 [8, 15]	**0.0097**
*Glasgow* depression score categories^*^				
Total score < 13	106 (46.5%)	110 (80.3%)	216 (59.2%)	**< 0.001**
Total score ≥ 13	122 (53.5%)	27 (19.7%)	149 (40.8%)	
*Disease characteristics of DSRD patients*				
Movement disorder	160 (70.2%)			
Altered mental status	162 (71.1%)			
Cognitive decline	112 (49.1%)			
Focal neurologic deficits/seizure	11 (4.8%)			
Insomnia	150 (65.8%)			
Language deficits	159 (69.7%)			
Psychiatric symptoms	153 (67.1%)			
Total symptoms	4.98 (1.51			
*Neurological Conditions of DSN Cohort*				
Autism Spectrum Disorder (ASD)		69 (51%)		
Epilepsy/Infantile Spasms		39 (28%)		
Moyamoya Vasculopathy/Stroke		16 (12%)		
Deafness		13 (9%)		

Data are frequency (%) or mean (SD). ^*^p < 0.05 (in bold font)

DSRD: Down syndrome regression disorder; DSN: Down syndrome with neurological disorders; PedsQL: Pediatric quality of life– Family impact module; HRQL: Health-related quality of life

† As defined by the United States Census Bureau

### Comparison of characteristics between cohorts

In the DSRD group, individuals were, on average, older than those in the DSN group (20.1 years [SD = 7.4] vs. 16.1 years [SD = 5.3], *p* < 0.001), while the groups were comparable in terms of sex, ethnicity, race, and duration of symptoms (Table 1).

Caregivers of individuals with DSRD reported greater burden than those caring for individuals with DSN (Table 2). Notably, DSRD caregivers were more likely to experience increased financial burden (65% vs. 49.6%, *p* = 0.003), housing changes (23.1% vs. 13.1%, *p* = 0.02), disrupted sleep (77.6% vs. 22.6%, *p* < 0.001), negative impacts on social networks (71.1% vs. 34.3%, *p* < 0.001), and worsened mental health (77.5% vs. 21.2%, *p* < 0.001). Detailed survey responses for PedsQL, GDS, and ZCB by DSRD and DSN groups are presented in Supplementary Tables 1–3. Responses to questions on primary positive support networks before and after a child’s/relative’s diagnosis, as listed the CDS, are provided in Supplementary Table 4. Caregivers of individuals with DSRD had lower scores for positive support networks compared to those caring for individuals with DSN, when comparing pre- to post-diagnosis (mean difference [95% CI]: -0.9 [-1.2, -0.5)]. In particular, DSRD caregivers reported an average change in positive support networks of -1.3 [-1.5, -1.1], while DSN caregivers reported − 0.4 [-0.7, -0.1], indicating greater declines in perceived support post-diagnosis for the DSRD group.

**Table 2:** Responses of CDS items by DSRD and DSN caregiver groups

	DSRD	DSN	Total	*p*
	(*n* = 228)	(*n* = 137)	(*n* = 365)	
*Financial impact*				
Modify work hours				
No	26 (11.5%)	90 (65.7%)	116 (32.0%)	**< 0.001**
Yes	200 (88.5%)	47 (34.3%)	247 (68.0%)	
Stop working				
No	109 (48.7%)	98 (71.5%)	207 (57.3%)	**< 0.001**
Yes	115 (51.3%)	39 (28.5%)	154 (42.7%)	
Increased financial burden				
No	78 (34.5%)	69 (50.4%)	147 (40.5%)	**0.003**
Yes	148 (65.5%)	68 (49.6%)	216 (59.5%)	
Modify educational/career plans				
No	124 (56.1%)	88 (64.2%)	212 (59.2%)	0.128
Yes	97 (43.9%)	49 (35.8%)	146 (40.8%)	
*Housing*				
Change housing				
No	173 (76.9%)	119 (86.9%)	292 (80.7%)	**0.020**
Yes	52 (23.1%)	18 (13.1%)	70 (19.3%)	
Move closer to medical care				
No	210 (94.2%)	134 (97.8%)	344 (95.6%)	0.121
Yes	13 (5.8%)	3 (2.2%)	16 (4.4%)	
Landlord issues				
No	205 (90.7%)	135 (98.5%)	340 (93.7%)	**0.003**
Yes	21 (9.3%)	2 (1.5%)	23 (6.3%)	
Law enforcement involvement				
No	202 (89.4%)	136 (99.3%)	338 (93.1%)	**< 0.001**
Yes	24 (10.6%)	1 (0.7%)	25 (6.9%)	
*Sleep*				
Changed sleeping arrangements				
No	83 (36.9%)	101 (73.7%)	184 (50.8%)	**< 0.001**
Yes	142 (63.1%)	36 (26.3%)	178 (49.2%)	
Less sleep				
No	99 (43.4%)	117 (85.4%)	216 (59.2%)	**< 0.001**
Yes	129 (56.6%)	20 (14.6%)	149 (40.8%)	
Sleep shifts				
No	187 (82.0%)	130 (94.9%)	317 (86.8%)	**< 0.001**
Yes	41 (18.0%)	7 (5.1%)	48 (13.2%)	
Changed sleep hours to match				
No	149 (65.4%)	116 (84.7%)	265 (72.6%)	**< 0.001**
Yes	79 (34.6%)	21 (15.3%)	100 (27.4%)	
Other change				
No	200 (87.7%)	137 (100.0%)	337 (92.3%)	**< 0.001**
Yes	28 (12.3%)	0 (0.0%)	28 (7.7%)	
Worse sleep quality				
No	23 (10.2%)	89 (65.0%)	112 (30.9%)	**< 0.001**
Yes	203 (89.8%)	48 (35.0%)	251 (69.1%)	
Disrupted sleep				
No	51 (22.4%)	106 (77.4%)	157 (43.0%)	**< 0.001**
Yes	177 (77.6%)	31 (22.6%)	208 (57.0%)	
Fragmented sleep				
No	108 (47.4%)	102 (74.5%)	210 (57.5%)	**< 0.001**
Yes	120 (52.6%)	35 (25.5%)	155 (42.5%)	
Not rested				
No	67 (29.4%)	122 (89.1%)	189 (51.8%)	**< 0.001**
Yes	161 (70.6%)	15 (10.9%)	176 (48.2%)	
Nap during day				
No	142 (62.3%)	128 (93.4%)	270 (74.0%)	**< 0.001**
Yes	86 (37.7%)	9 (6.6%)	95 (26.0%)	
Other negative changes				
No	218 (95.6%)	134 (97.8%)	352 (96.4%)	0.385
Yes	10 (4.4%)	3 (2.2%)	13 (3.6%)	
*Social networks and relationships*				
Quality of adult friendships impacted				
Positive or no change	80 (35.6%)	97 (70.8%)	177 (48.9%)	**< 0.001**
Negative change	145 (64.4%)	40 (29.2%)	185 (51.1%)	
Social networks impacted				
Positive or no change	65 (28.9%)	90 (65.7%)	155 (42.8%)	**< 0.001**
Negative change	160 (71.1%)	47 (34.3%)	207 (57.2%)	
Marriage impacted				
Positive or no change	107 (48.0%)	110 (80.3%)	217 (60.3%)	**< 0.001**
Negative change	116 (52.0%)	27 (19.7%)	143 (39.7%)	
Size of friend network changed				
Positive or no change	102 (45.9%)	85 (62.0%)	187 (52.1%)	**0.003**
Negative change	120 (54.1%)	52 (38.0%)	172 (47.9%)	
*Mental health*				
Worsened mental health				
No	50 (22.5%)	108 (78.8%)	158 (44.0%)	**< 0.001**
Yes	172 (77.5%)	29 (21.2%)	201 (56.0%)	
Frustration/anger towards loved one				
No	104 (46.2%)	126 (92.0%)	230 (63.5%)	**< 0.001**
Yes	121 (53.8%)	11 (8.0%)	132 (36.5%)	
Fears about child’s future or family’s future				
No	13 (5.8%)	44 (32.1%)	57 (15.8%)	**< 0.001**
Yes	211 (94.2%)	93 (67.9%)	304 (84.2%)	
Fears about misdiagnosis				
No	71 (32.1%)	113 (82.5%)	184 (51.4%)	**< 0.001**
Yes	150 (67.9%)	24 (17.5%)	174 (48.6%)	
Fears about access to treatment				
No	34 (15.4%)	75 (54.7%)	109 (30.4%)	**< 0.001**
Yes	187 (84.6%)	62 (45.3%)	249 (69.6%)	

Data are frequency (%). ^*^p < 0.05 shown in bold. DSRD: Down syndrome regression disorder; DSN: Down syndrome with neurological disorders; CDS: Caregiver distress survey. The frequency and percent of incomplete variables: Modify work hours (*n* = 2, 0.55%); Stop working (*n* = 4, 1.1%); Increased financial burden (*n* = 2, 0.55%); Modify educational/career plans (*n* = 7, 1.9%); Change housing (*n* = 3, 0.8%); Move closer to medical care (*n* = 5, 1.4%); Landlord issues (*n* = 2, 0.55%); Law enforcement involvement (*n* = 2, 0.55%); Changed sleeping arrangements (*n* = 3, 0.8%); Worse sleep quality (*n* = 2, 0.55%); Quality of adult friendships impacted (*n* = 3, 0.8%); Social networks impacted (*n* = 3, 0.8%); Marriage impacted (*n* = 5, 1.4%); Size of friend network changed (*n* = 6, 1.6%); Worsened mental health (*n* = 6, 1.6%); Frustration/anger towards loved one (*n* = 3, 0.8%); Fears about child’s future or family’s future (*n* = 4, 1.1%); Fears about misdiagnosis (*n* = 7, 1.9%); Fears about access to treatment (*n* = 7, 1.9%)

### Quality of life, distress, and care burden in DSRD and DSN

**Fig. 1:**
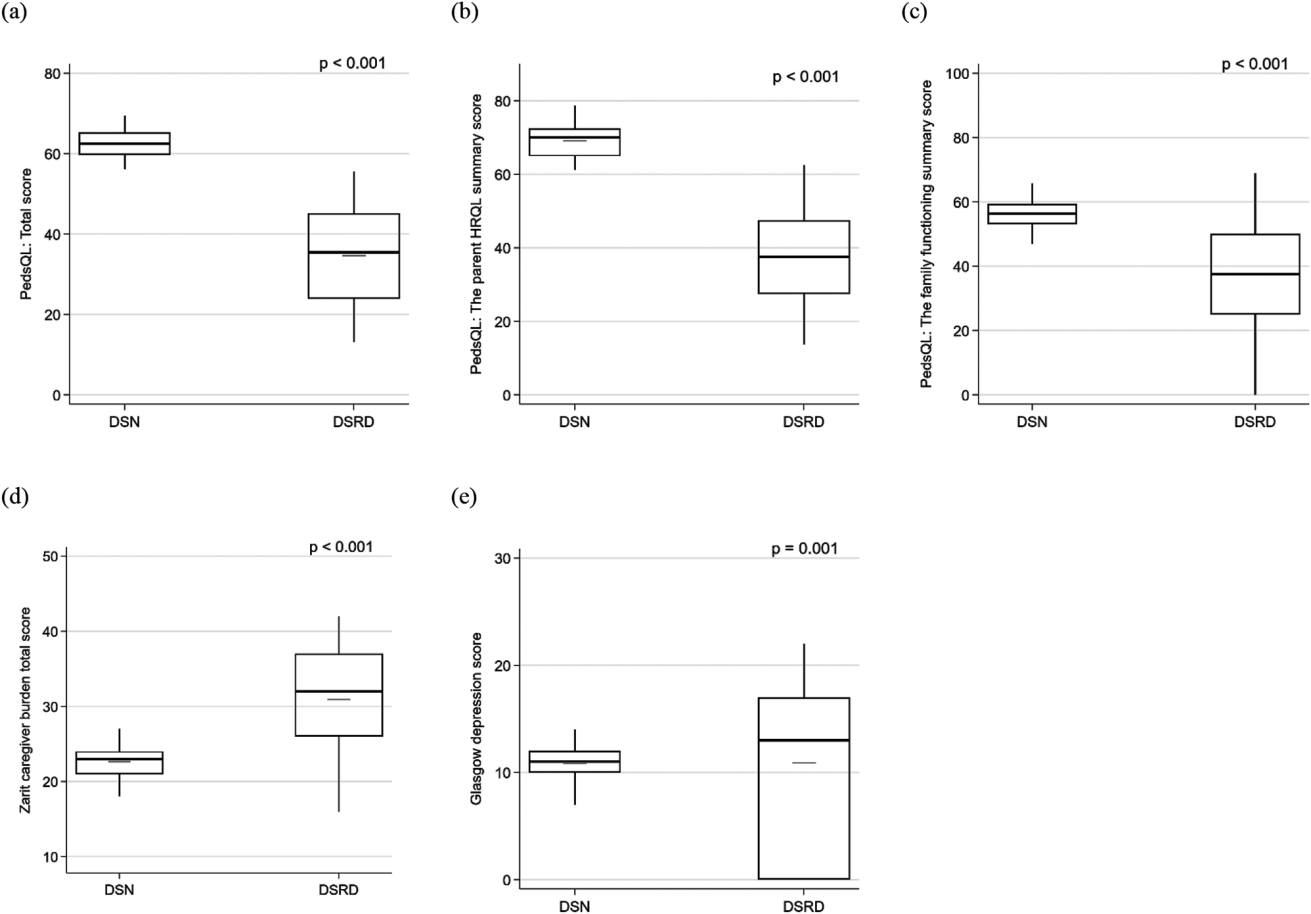
Distributions of PedsQL total score, parent HRQL summary score, PedsQL family functioning summary score, ZCB, and GDS are shown via box-and-whisker plots across DSRD and DSN caregiver groups. Each plot summarizes minimum, lower quantile, median (bold line), upper quantile, and maximum, along with mean shown in gray line. DSRD: Down syndrome regression disorder; DSN: Down syndrome with neurological disorders; PedsQL: Pediatric quality of life– Family impact module; HRQL: Health-related quality of life; ZCB: Zarit caregiver burden; and GDS: Glasgow depression scale

**Fig. 2:**
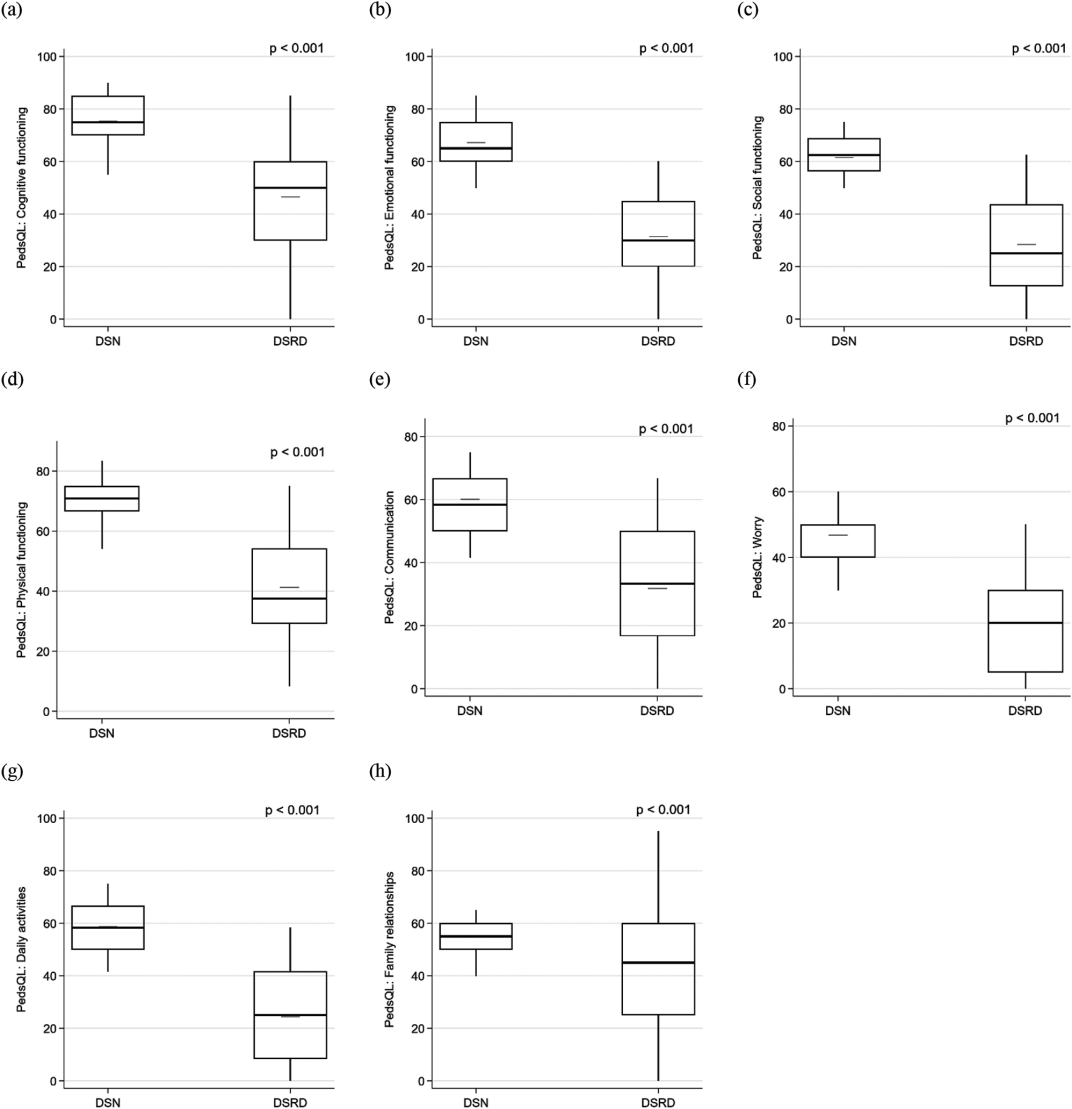
Distributions of PedsQL subdomains are shown via box-and-whisker plots across DSRD and DSN caregiver groups. Each plot summarizes minimum, lower quantile, median (bold line), upper quantile, and maximum, along with mean shown in gray line. DSRD: Down syndrome regression disorder; DSN: Down syndrome with neurological disorders; and PedsQL: Pediatric quality of life– Family impact module

Caregivers of individuals with DSRD reported lower quality of life in the total PedsQL, parent HRQL summary, and family functioning summary scores (Fig. 1), as well as across all eight PedsQL domains (Fig. 2) compared to caregivers of those with DSN (Table 1). Specifically, the average reduction in total PedsQL score for DSRD caregivers was − 27.9 [-30.2, -25.5], in the parent HRQL summary score − 31.6 [-34.3, -28.9], and in the family functioning summary score − 19.1 [-22.4, -15.9], reflecting a more substantial impact on overall well-being and daily functioning (Table 3). DSRD caregivers also had higher median GDS (Fig. 1; Table 1) than DSN caregivers, with a median difference of 2 [0.7, 3.4] (Table 3). They were also more likely to meet clinical depression criteria (*p* < 0.001, Table 1), with higher odds of a GDS score ≥ 13 (odds ratio (OR) [95% CI]: 4.7 [2.9, 7.7]; Table 3). Furthermore, DSRD caregivers reported higher ZCB scores compared to DSN caregivers (Fig. 1; Table 1), with an average increase of 8.3 [6.3, 9.7] (Table 3).

**Table 3:** Estimated regression coefficients with 95% confidence intervals (CIs) for PedsQL and ZCB, and GDS scores, and odds ratios (ORs) with 95% CI for GDS (≥ 13 vs. < 13) across DSRD and DSN caregiver groups

	Mean Difference	SE	[95% CI]	*p*
*PedsQL*				
Physical functioning score				
DSN	0.0	0.0		
DSRD	-29.39	1.76	[-32.85, -25.92]	**< 0.001**
Emotional functioning score				
DSN	0.0	0.0		
DSRD	-35.76	1.69	[-39.09, -32.43]	**< 0.001**
Social functioning score				
DSN	0.0	0.0		
DSRD	-33.02	1.90	[-36.76, -29.28]	**< 0.001**
Cognitive functioning score				
DSN	0.0	0.0		
DSRD	-28.91	2.04	[-32.93, -24.89]	**< 0.001**
Communication score				
DSN	0.0	0.0		
DSRD	-28.24	1.88	[-31.93, -24.55]	**< 0.001**
Worry score				
DSN	0.0	0.0		
DSRD	-26.75	1.60	[-29.91, -23.60]	**< 0.001**
Daily activities score				
DSN	0.0	0.0		
DSRD	-34.22	1.79	[-37.75, -30.69]	**< 0.001**
Family relationships score				
DSN	0.0	0.0		
DSRD	-10.09	2.19	[-14.39, -5.80]	**< 0.001**
Total score				
DSN	0.0	0.0		
DSRD	-27.87	1.20	[-30.23, -25.52]	**< 0.001**
The parent HRQL summary score				
DSN	0.0	0.0		
DSRD	-31.59	1.36	[-34.26, -28.92]	**< 0.001**
The family functioning summary score				
DSN	0.0	0.0		
DSRD	-19.14	1.67	[-22.43, -15.86]	**< 0.001**
* Zarit caregiver burden total score*				
DSN	0.0	0.0		
DSRD	8.30	0.70	[6.93, 9.67]	**< 0.001**
	Median Difference	SE	[95% CI]	*p*
*Glasgow depression score*				
DSN	0.0	0.0		
DSRD	2.00	0.69	[0.65, 3.35]	**0.004**
	OR	SE	[95% CI]	*p*
*Glasgow depression score (≥ 13 vs. < 13)*				
DSN	1.0	0.0		
DSRD	4.69	1.18	[2.86, 7.69]	**< 0.001**

^*^p < 0.05 (in bold font). DSRD: Down syndrome regression disorder; DSN: Down syndrome with neurological disorders; PedsQL: Pediatric quality of life– Family impact module; HRQL: Health-related quality of life; ZCB: Zarit caregiver burden; and GDS: Glasgow depression scale

Further analyses (Supplementary Tables 5 and Supplementary Fig. 1) illustrated that among DSRD caregivers, a negative change in social networks was associated with lower mean levels on PedsQL total score (-6.7 [-11.6, -1.7]), parent HRQL summary score (-7.3 [-13.0, -1.7]), and family functioning summary score (-8.2 [-15.0, -1.3]), as well as a higher mean ZCB score (3.0 [0.12, 6.0]) compared to the DSN group. Similarly, worsened mental health was linked with lower mean levels on PedsQL total score (-10.5 [-16.0, -5.0]), parent HRQL summary score (-11.7 [-18.0, -5.5]), and family functioning summary score (-11.5 [-19.4, -3.6]), and with a higher mean ZCB score (6.6 [3.4, 9.8]) among DSRD caregivers compared to DSN caregivers.

Financial burden was also associated with reduced mean PedsQL total score (-5.0 [-9.7, -0.3]) and family functioning summary score (-9.0 [-15.6, -2.5]), along with higher mean ZCB (4.9 [2.2, 7.6]) and median GDS (5.0 [1.8, 8.2]) scores in DSRD caregivers compared to DSN caregivers. Disrupted sleep among DSRD caregivers was linked to lower mean PedsQL total (-6.3 [-11.9, -0.7]) and parent HRQL summary (-6.9 [-13.2, -0.6]) scores compared to DSN caregivers. Supplementary Fig. 1 additionally demonstrates significant associations between PedsQL domains and potential DSRD CDS factors within the DSRD and DSN groups. Finally, being male appeared to be associated with lower odds of clinical depression among DSRD caregivers compared to DSN caregivers (OR: 0.2 [0.1, 0.5]).

## Discussion

Our study revealed significant differences in caregiver burden and mental health impacts between caregivers of individuals with DSRD and those caring for individuals with DSN. Caregivers of individuals with DSRD experienced greater challenges across multiple domains, including financial strain, housing stability, sleep disruption, social support, and mental health. These caregivers demonstrated a lower quality of life on the PedsQL, greater levels of burden on the ZBS, and a higher prevalence of depression symptoms on the GDS compared to DSN caregivers. Overall, this study underscores the profound level of distress and burden faced by caregivers of individuals with DSRD.

The impact of caregiver burden in this study was multimodal, as evidenced by elevated scores not only on the ZBS but also across the PedsQL, GDS, and DSRD CDS, indicating a diffuse interruption in caregiver life. One possible explanation for this finding is inherent differences in symptoms between DSRD and DSN. Symptoms of DSRD are a combination of catatonia and neuropsychiatric disease, which not only impairs functional aspects of life but also disrupts social engagement [2]. In contrast, individuals with DSN primarily experience focal neurologic deficits and/or transient episodes of neurologic deficit (e.g., seizure). In DSRD, symptoms such as severe mood disturbances, anxiety, and loss of previously acquired skills impair functional abilities, including mobility and cognitive processing, and also interfere profoundly with social engagement and emotional regulation [1, 2]. These symptoms require caregivers to provide constant, dynamic support that addresses both physical and behavioral needs, a demand that is less pronounced in the DSN group. The authors propose that the neuropsychiatric symptoms in DSRD serve as the primary driver of this multimodal impact, which could explain the intensified burden on DSRD caregivers across all metrics measured in this study.

Existing literature suggests that caregivers of individuals with DSN experience higher levels of burden compared to those caring for neurotypical individuals [10, 18]. For example, caregivers of individuals with ASD experience increased stress due to intensive care demands, behavioral challenges, and the societal stigma associated with ASD [19, 20]. Similarly, caregivers of individuals with stroke report substantial burden due to the sudden onset of care responsibilities, the physical demands of caregiving, and the long-term nature of stroke recovery [21, 22]. Further, caregivers of individuals with epilepsy face unique stressors related to the unpredictability of seizures, emotional strain, and the need for constant vigilance, all contributing to elevated caregiver burden [23]. However, our study found that caregivers of individuals with DSRD reported even higher levels of burden than those caring for individuals with DSN, highlighting the profound challenges faced by this unique population.

Initially, it was anticipated that demographic differences, such as lower socioeconomic status (SES) and certain ethnicity backgrounds, would link with greater caregiver burden, as prior research has suggested that lower SES often is related to increased caregiver distress [24]. However, this was not observed in our study. One potential explanation is that DSRD, as a relatively new diagnosis, may primarily be accessible to families with greater resources who can seek out the specialized care required for diagnosis. Literature demonstrates that families with lower SES often encounter challenges obtaining timely diagnosis for rare genetic diseases, due to difficulties in obtaining relevant referrals and delays in care, a phenomenon that may extend to.

DSRD [25]. Further, DSRD specialists are often only located at academic medical centers across the country, which may be less accessible to lower SES families. Consequently, families of lower SES may be underrepresented in this study due to limited access to DSRD diagnostic and treatment services. Another possible explanation is that the intense challenges of managing DSRD symptoms may overshadow external influences, creating a level of burden that is consistently high across demographic groups. The pervasive stress of caring for individuals with DSRD could establish a baseline level of caregiver burden that remains relatively unaffected by socioeconomic variations such as income or education.

This study is not without limitations, as it is an initial investigation into the impacts of DSRD on caregivers. Bias may have affected our findings. Firstly, severity bias may have impacted the results, as caregivers of individuals with more severe DSRD symptoms might have been more likely to participate, potentially skewing the results toward higher perceived burdens. Additionally, ascertainment bias could arise since the caregiver burden measures were self-reported and perception of caregiving responsibilities may differ among participants. The study also relied on retrospective reflection, which could introduce cognitive bias, leading participants to misremember, misinterpret, or overinterpret their caregiving experiences. Furthermore, selection bias could be present, as the patient population was recruited from a DSRD Facebook support group, potentially limiting the demographic diversity and socioeconomic representation. Frequent participation in surveys over the past few years might have contributed to survey fatigue, lowering enrollment rates and affecting the sample size and diversity. In addition, the DSN cohort was composed of a heterogenous group of conditions which affect individuals with DS. While these conditions did not statistically differ with regards to responses in this study, further expansion and comparison to non-DS cohorts (e.g., individuals with epilepsy without DS) will be beneficial. Finally, the accuracy of the cohort may have been affected by the inability to confirm both DSRD and DSN diagnoses on a medical basis. These limitations may affect the generalizability of the findings.

## Conclusions

In summary, this study reveals a concerning theme wherein caregivers of individuals with DSRD experience a significantly greater burden compared to caregivers of those with DSN. These findings highlight the need for a deeper understanding of the unique challenges faced by caregivers of individuals with DSRD and the urgent need for targeted support.

## Electronic supplementary material

Below is the link to the electronic supplementary material.

Supplementary Material 1

Supplementary Material 2

Supplementary Material 3

Supplementary Material 4

Supplementary Material 5

Supplementary Material 6

## Data Availability

Anonymized data is available upon request to qualified researchers pending IRB approval.

## Abbreviations

ASD: Autism spectrum disorder
CDS: Caregiver Distress Survey
DS: Down syndrome
DSRD: Down Syndrome Regression Disorder
DSN: Down syndrome other neurologic disorders
GDS: Glassglow Depression Scale
HRQL: Health-Related Quality of Life
PedsQL: Pediatric Quality of Life Impact Module
SES: Socioeconomic status
ZCB: Zarit Caregiver Burden Assessment
