# Supplementary material for: Caregiver burden and familial impact in Down Syndrome Regression Disorder
Source: Orphanet J Rare Dis. 2025 Mar 14;20:126. doi: 10.1186/s13023-025-03644-0 (PMC11909950; doi:10.1186/s13023-025-03644-0)
Supplement: Supplementary file 1 — Supplementary Material 1 [file 13023_2025_3644_MOESM1_ESM.docx]

| **Table S1**. PedsQL subdomains survey responses across DSRD and DSN caregiver groups. | | | |
| --- | --- | --- | --- |
|  | DSRD | DSN | Total |
|  | (n = 228) | (n = 137) | (n = 365) |
| *Physical functioning* |  |  |  |
| Tired during day |  |  |  |
| Almost always | 85 (37.3%) | 2 (1.5%) | 87 (23.8%) |
| Often | 89 (39.0%) | 13 (9.5%) | 102 (27.9%) |
| Sometimes | 49 (21.5%) | 48 (35.0%) | 97 (26.6%) |
| Almost never | 5 (2.2%) | 48 (35.0%) | 53 (14.5%) |
| Never | 0 (0.0%) | 26 (19.0%) | 26 (7.1%) |
| Tired in morning |  |  |  |
| Almost always | 74 (32.5%) | 1 (0.7%) | 75 (20.5%) |
| Often | 76 (33.3%) | 9 (6.6%) | 85 (23.3%) |
| Sometimes | 63 (27.6%) | 64 (46.7%) | 127 (34.8%) |
| Almost never | 14 (6.1%) | 54 (39.4%) | 68 (18.6%) |
| Never | 1 (0.4%) | 9 (6.6%) | 10 (2.7%) |
| Tired to do things I like |  |  |  |
| Almost always | 56 (24.6%) | 0 (0.0%) | 56 (15.3%) |
| Often | 91 (39.9%) | 8 (5.8%) | 99 (27.1%) |
| Sometimes | 63 (27.6%) | 70 (51.1%) | 133 (36.4%) |
| Almost never | 18 (7.9%) | 45 (32.8%) | 63 (17.3%) |
| Never | 0 (0.0%) | 14 (10.2%) | 14 (3.8%) |
| Get headache |  |  |  |
| Almost always | 19 (8.3%) | 0 (0.0%) | 19 (5.2%) |
| Often | 35 (15.4%) | 3 (2.2%) | 38 (10.4%) |
| Sometimes | 74 (32.5%) | 13 (9.5%) | 87 (23.8%) |
| Almost never | 57 (25.0%) | 55 (40.1%) | 112 (30.7%) |
| Never | 43 (18.9%) | 66 (48.2%) | 109 (29.9%) |
| Feel weak |  |  |  |
| Almost always | 23 (10.1%) | 0 (0.0%) | 23 (6.3%) |
| Often | 51 (22.4%) | 16 (11.7%) | 67 (18.4%) |
| Sometimes | 90 (39.5%) | 51 (37.2%) | 141 (38.6%) |
| Almost never | 29 (12.7%) | 42 (30.7%) | 71 (19.5%) |
| Never | 35 (15.4%) | 28 (20.4%) | 63 (17.3%) |
| Feel sick to my stomach |  |  |  |
| Almost always | 6 (2.6%) | 0 (0.0%) | 6 (1.6%) |
| Often | 47 (20.6%) | 1 (0.7%) | 48 (13.2%) |
| Sometimes | 69 (30.3%) | 14 (10.2%) | 83 (22.7%) |
| Almost never | 56 (24.6%) | 39 (28.5%) | 95 (26.0%) |
| Never | 50 (21.9%) | 83 (60.6%) | 133 (36.4%) |
| *Emotional functioning* |  |  |  |
| Anxious |  |  |  |
| Almost always | 59 (25.9%) | 2 (1.5%) | 61 (16.7%) |
| Often | 90 (39.5%) | 15 (10.9%) | 105 (28.8%) |
| Sometimes | 65 (28.5%) | 54 (39.4%) | 119 (32.6%) |
| Almost never | 14 (6.1%) | 59 (43.1%) | 73 (20.0%) |
| Never | 0 (0.0%) | 7 (5.1%) | 7 (1.9%) |
| Sad |  |  |  |
| Almost always | 65 (28.5%) | 1 (0.7%) | 66 (18.1%) |
| Often | 82 (36.0%) | 17 (12.4%) | 99 (27.1%) |
| Sometimes | 72 (31.6%) | 53 (38.7%) | 125 (34.2%) |
| Almost never | 8 (3.5%) | 39 (28.5%) | 47 (12.9%) |
| Never | 1 (0.4%) | 27 (19.7%) | 28 (7.7%) |
| Angry |  |  |  |
| Almost always | 32 (14.0%) | 0 (0.0%) | 32 (8.8%) |
| Often | 59 (25.9%) | 2 (1.5%) | 61 (16.7%) |
| Sometimes | 97 (42.5%) | 15 (10.9%) | 112 (30.7%) |
| Almost never | 32 (14.0%) | 73 (53.3%) | 105 (28.8%) |
| Never | 8 (3.5%) | 47 (34.3%) | 55 (15.1%) |
| Frustrated |  |  |  |
| Almost always | 78 (34.2%) | 0 (0.0%) | 78 (21.4%) |
| Often | 89 (39.0%) | 0 (0.0%) | 89 (24.4%) |
| Sometimes | 53 (23.2%) | 70 (51.1%) | 123 (33.7%) |
| Almost never | 7 (3.1%) | 58 (42.3%) | 65 (17.8%) |
| Never | 1 (0.4%) | 9 (6.6%) | 10 (2.7%) |
| Hopeless |  |  |  |
| Almost always | 58 (25.4%) | 1 (0.7%) | 59 (16.2%) |
| Often | 66 (28.9%) | 9 (6.6%) | 75 (20.5%) |
| Sometimes | 67 (29.4%) | 45 (32.8%) | 112 (30.7%) |
| Almost never | 32 (14.0%) | 51 (37.2%) | 83 (22.7%) |
| Never | 5 (2.2%) | 31 (22.6%) | 36 (9.9%) |
| *Social functioning* |  |  |  |
| Isolated from others |  |  |  |
| Almost always | 71 (31.1%) | 1 (0.7%) | 72 (19.7%) |
| Often | 82 (36.0%) | 6 (4.4%) | 88 (24.1%) |
| Sometimes | 58 (25.4%) | 60 (43.8%) | 118 (32.3%) |
| Almost never | 11 (4.8%) | 59 (43.1%) | 70 (19.2%) |
| Never | 6 (2.6%) | 11 (8.0%) | 17 (4.7%) |
| Trouble with support from others |  |  |  |
| Almost always | 57 (25.0%) | 1 (0.7%) | 58 (15.9%) |
| Often | 81 (35.5%) | 12 (8.8%) | 93 (25.5%) |
| Sometimes | 64 (28.1%) | 52 (38.0%) | 116 (31.8%) |
| Almost never | 19 (8.3%) | 62 (45.3%) | 81 (22.2%) |
| Never | 7 (3.1%) | 10 (7.3%) | 17 (4.7%) |
| No time for social activities |  |  |  |
| Almost always | 90 (39.5%) | 0 (0.0%) | 90 (24.7%) |
| Often | 71 (31.1%) | 14 (10.2%) | 85 (23.3%) |
| Sometimes | 56 (24.6%) | 64 (46.7%) | 120 (32.9%) |
| Almost never | 9 (3.9%) | 53 (38.7%) | 62 (17.0%) |
| Never | 2 (0.9%) | 6 (4.4%) | 8 (2.2%) |
| No energy for social activities |  |  |  |
| Almost always | 68 (29.8%) | 1 (0.7%) | 69 (18.9%) |
| Often | 74 (32.5%) | 11 (8.0%) | 85 (23.3%) |
| Sometimes | 65 (28.5%) | 61 (44.5%) | 126 (34.5%) |
| Almost never | 18 (7.9%) | 56 (40.9%) | 74 (20.3%) |
| Never | 3 (1.3%) | 8 (5.8%) | 11 (3.0%) |
| *Cognitive functioning* |  |  |  |
| Hard to keep attention |  |  |  |
| Almost always | 29 (12.7%) | 1 (0.7%) | 30 (8.2%) |
| Often | 76 (33.3%) | 9 (6.6%) | 85 (23.3%) |
| Sometimes | 82 (36.0%) | 31 (22.6%) | 113 (31.0%) |
| Almost never | 39 (17.1%) | 51 (37.2%) | 90 (24.7%) |
| Never | 2 (0.9%) | 45 (32.8%) | 47 (12.9%) |
| Hard to remember what people tell me |  |  |  |
| Almost always | 21 (9.2%) | 0 (0.0%) | 21 (5.8%) |
| Often | 67 (29.4%) | 6 (4.4%) | 73 (20.0%) |
| Sometimes | 77 (33.8%) | 39 (28.5%) | 116 (31.8%) |
| Almost never | 55 (24.1%) | 60 (43.8%) | 115 (31.5%) |
| Never | 8 (3.5%) | 32 (23.4%) | 40 (11.0%) |
| Hard to remember what I heard |  |  |  |
| Almost always | 18 (7.9%) | 0 (0.0%) | 18 (4.9%) |
| Often | 59 (25.9%) | 3 (2.2%) | 62 (17.0%) |
| Sometimes | 78 (34.2%) | 42 (30.7%) | 120 (32.9%) |
| Almost never | 54 (23.7%) | 61 (44.5%) | 115 (31.5%) |
| Never | 19 (8.3%) | 31 (22.6%) | 50 (13.7%) |
| Hard to think quick |  |  |  |
| Almost always | 15 (6.6%) | 0 (0.0%) | 15 (4.1%) |
| Often | 62 (27.2%) | 0 (0.0%) | 62 (17.0%) |
| Sometimes | 85 (37.3%) | 15 (10.9%) | 100 (27.4%) |
| Almost never | 50 (21.9%) | 57 (41.6%) | 107 (29.3%) |
| Never | 16 (7.0%) | 65 (47.4%) | 81 (22.2%) |
| Trouble remembering what I was thinking |  |  |  |
| Almost always | 15 (6.6%) | 0 (0.0%) | 15 (4.1%) |
| Often | 63 (27.6%) | 3 (2.2%) | 66 (18.1%) |
| Sometimes | 88 (38.6%) | 32 (23.4%) | 120 (32.9%) |
| Almost never | 49 (21.5%) | 60 (43.8%) | 109 (29.9%) |
| Never | 13 (5.7%) | 42 (30.7%) | 55 (15.1%) |
| *Communication* |  |  |  |
| Others don't understand |  |  |  |
| Almost always | 118 (51.8%) | 0 (0.0%) | 118 (32.3%) |
| Often | 65 (28.5%) | 14 (10.2%) | 79 (21.6%) |
| Sometimes | 41 (18.0%) | 63 (46.0%) | 104 (28.5%) |
| Almost never | 4 (1.8%) | 58 (42.3%) | 62 (17.0%) |
| Never | 0 (0.0%) | 2 (1.5%) | 2 (0.5%) |
| Hard to talk about relative's health with others |  |  |  |
| Almost always | 52 (22.8%) | 2 (1.5%) | 54 (14.8%) |
| Often | 68 (29.8%) | 8 (5.8%) | 76 (20.8%) |
| Sometimes | 68 (29.8%) | 65 (47.4%) | 133 (36.4%) |
| Almost never | 31 (13.6%) | 56 (40.9%) | 87 (23.8%) |
| Never | 9 (3.9%) | 6 (4.4%) | 15 (4.1%) |
| Hard to tell doctors how I feel |  |  |  |
| Almost always | 38 (16.7%) | 0 (0.0%) | 38 (10.4%) |
| Often | 72 (31.6%) | 9 (6.6%) | 81 (22.2%) |
| Sometimes | 67 (29.4%) | 60 (43.8%) | 127 (34.8%) |
| Almost never | 32 (14.0%) | 66 (48.2%) | 98 (26.8%) |
| Never | 19 (8.3%) | 2 (1.5%) | 21 (5.8%) |
| *Worry* |  |  |  |
| Whether treatments are working |  |  |  |
| Almost always | 108 (47.4%) | 6 (4.4%) | 114 (31.2%) |
| Often | 80 (35.1%) | 45 (32.8%) | 125 (34.2%) |
| Sometimes | 31 (13.6%) | 43 (31.4%) | 74 (20.3%) |
| Almost never | 7 (3.1%) | 42 (30.7%) | 49 (13.4%) |
| Never | 2 (0.9%) | 1 (0.7%) | 3 (0.8%) |
| About side effects of medications/treatments |  |  |  |
| Almost always | 99 (43.4%) | 6 (4.4%) | 105 (28.8%) |
| Often | 79 (34.6%) | 45 (32.8%) | 124 (34.0%) |
| Sometimes | 41 (18.0%) | 47 (34.3%) | 88 (24.1%) |
| Almost never | 8 (3.5%) | 37 (27.0%) | 45 (12.3%) |
| Never | 1 (0.4%) | 2 (1.5%) | 3 (0.8%) |
| Others react to condition |  |  |  |
| Almost always | 91 (39.9%) | 3 (2.2%) | 94 (25.8%) |
| Often | 69 (30.3%) | 47 (34.3%) | 116 (31.8%) |
| Sometimes | 43 (18.9%) | 50 (36.5%) | 93 (25.5%) |
| Almost never | 13 (5.7%) | 34 (24.8%) | 47 (12.9%) |
| Never | 12 (5.3%) | 3 (2.2%) | 15 (4.1%) |
| How illness is affecting other family members |  |  |  |
| Almost always | 97 (42.5%) | 0 (0.0%) | 97 (26.6%) |
| Often | 70 (30.7%) | 54 (39.4%) | 124 (34.0%) |
| Sometimes | 40 (17.5%) | 44 (32.1%) | 84 (23.0%) |
| Almost never | 14 (6.1%) | 37 (27.0%) | 51 (14.0%) |
| Never | 7 (3.1%) | 2 (1.5%) | 9 (2.5%) |
| Future |  |  |  |
| Almost always | 159 (69.7%) | 8 (5.8%) | 167 (45.8%) |
| Often | 49 (21.5%) | 49 (35.8%) | 98 (26.8%) |
| Sometimes | 19 (8.3%) | 50 (36.5%) | 69 (18.9%) |
| Almost never | 1 (0.4%) | 29 (21.2%) | 30 (8.2%) |
| Never | 0 (0.0%) | 1 (0.7%) | 1 (0.3%) |
| *Daily activities* |  |  |  |
| More time and effort |  |  |  |
| Almost always | 120 (52.6%) | 4 (2.9%) | 124 (34.0%) |
| Often | 68 (29.8%) | 31 (22.6%) | 99 (27.1%) |
| Sometimes | 32 (14.0%) | 51 (37.2%) | 83 (22.7%) |
| Almost never | 7 (3.1%) | 44 (32.1%) | 51 (14.0%) |
| Never | 1 (0.4%) | 7 (5.1%) | 8 (2.2%) |
| No time to finish household tasks |  |  |  |
| Almost always | 74 (32.5%) | 5 (3.6%) | 79 (21.6%) |
| Often | 75 (32.9%) | 15 (10.9%) | 90 (24.7%) |
| Sometimes | 64 (28.1%) | 63 (46.0%) | 127 (34.8%) |
| Almost never | 11 (4.8%) | 45 (32.8%) | 56 (15.3%) |
| Never | 4 (1.8%) | 9 (6.6%) | 13 (3.6%) |
| Too tired to finish household tasks |  |  |  |
| Almost always | 66 (28.9%) | 0 (0.0%) | 66 (18.1%) |
| Often | 80 (35.1%) | 0 (0.0%) | 80 (21.9%) |
| Sometimes | 68 (29.8%) | 62 (45.3%) | 130 (35.6%) |
| Almost never | 13 (5.7%) | 65 (47.4%) | 78 (21.4%) |
| Never | 1 (0.4%) | 10 (7.3%) | 11 (3.0%) |
| *Family relationships* |  |  |  |
| Lack of communication between family |  |  |  |
| Almost always | 28 (12.3%) | 1 (0.7%) | 29 (7.9%) |
| Often | 72 (31.6%) | 13 (9.5%) | 85 (23.3%) |
| Sometimes | 85 (37.3%) | 66 (48.2%) | 151 (41.4%) |
| Almost never | 29 (12.7%) | 57 (41.6%) | 86 (23.6%) |
| Never | 14 (6.1%) | 0 (0.0%) | 14 (3.8%) |
| Conflicts between family members |  |  |  |
| Almost always | 26 (11.4%) | 0 (0.0%) | 26 (7.1%) |
| Often | 51 (22.4%) | 6 (4.4%) | 57 (15.6%) |
| Sometimes | 88 (38.6%) | 57 (41.6%) | 145 (39.7%) |
| Almost never | 45 (19.7%) | 74 (54.0%) | 119 (32.6%) |
| Never | 18 (7.9%) | 0 (0.0%) | 18 (4.9%) |
| Difficulty making decisions together |  |  |  |
| Almost always | 24 (10.5%) | 3 (2.2%) | 27 (7.4%) |
| Often | 60 (26.3%) | 54 (39.4%) | 114 (31.2%) |
| Sometimes | 82 (36.0%) | 39 (28.5%) | 121 (33.2%) |
| Almost never | 44 (19.3%) | 41 (29.9%) | 85 (23.3%) |
| Never | 18 (7.9%) | 0 (0.0%) | 18 (4.9%) |
| Difficulty solving family problems together |  |  |  |
| Almost always | 25 (11.0%) | 0 (0.0%) | 25 (6.8%) |
| Often | 59 (25.9%) | 46 (33.6%) | 105 (28.8%) |
| Sometimes | 75 (32.9%) | 55 (40.1%) | 130 (35.6%) |
| Almost never | 50 (21.9%) | 36 (26.3%) | 86 (23.6%) |
| Never | 19 (8.3%) | 0 (0.0%) | 19 (5.2%) |
| Stress/tension between family members |  |  |  |
| Almost always | 40 (17.5%) | 0 (0.0%) | 40 (11.0%) |
| Often | 61 (26.8%) | 9 (6.6%) | 70 (19.2%) |
| Sometimes | 81 (35.5%) | 58 (42.3%) | 139 (38.1%) |
| Almost never | 33 (14.5%) | 70 (51.1%) | 103 (28.2%) |
| Never | 13 (5.7%) | 0 (0.0%) | 13 (3.6%) |
| Data are frequency (%). DSRD: Down syndrome regression disorder; DSN: Down syndrome with neurological disorders; and PedsQL: Pediatric quality of life – Family impact module. | | | |
