# Supplementary material for: Caregiver burden and familial impact in Down Syndrome Regression Disorder
Source: Orphanet J Rare Dis. 2025 Mar 14;20:126. doi: 10.1186/s13023-025-03644-0 (PMC11909950; doi:10.1186/s13023-025-03644-0)
Supplement: Supplementary file 2 — Supplementary Material 2 [file 13023_2025_3644_MOESM2_ESM.docx]

| **Table S2**. ZCB survey responses across DSRD and DSN caregiver groups. | | | |
| --- | --- | --- | --- |
|  | DSRD | DSN | Total |
|  | (n = 228) | (n = 137) | (n = 365) |
| Don't have enough time for yourself |  |  |  |
| Never | 1 (0.4%) | 1 (0.7%) | 2 (0.5%) |
| Rarely | 5 (2.2%) | 46 (33.6%) | 51 (14.0%) |
| Sometimes | 49 (21.5%) | 65 (47.4%) | 114 (31.2%) |
| Frequently | 87 (38.2%) | 25 (18.2%) | 112 (30.7%) |
| Nearly always | 86 (37.7%) | 0 (0.0%) | 86 (23.6%) |
| Stressed between caring and meeting other responsibilities |  |  |  |
| Never | 0 (0.0%) | 1 (0.7%) | 1 (0.3%) |
| Rarely | 3 (1.3%) | 19 (13.9%) | 22 (6.0%) |
| Sometimes | 33 (14.5%) | 70 (51.1%) | 103 (28.2%) |
| Frequently | 98 (43.0%) | 29 (21.2%) | 127 (34.8%) |
| Nearly always | 94 (41.2%) | 18 (13.1%) | 112 (30.7%) |
| Angry around relative |  |  |  |
| Never | 40 (17.5%) | 6 (4.4%) | 46 (12.6%) |
| Rarely | 71 (31.1%) | 63 (46.0%) | 134 (36.7%) |
| Sometimes | 83 (36.4%) | 47 (34.3%) | 130 (35.6%) |
| Frequently | 23 (10.1%) | 21 (15.3%) | 44 (12.1%) |
| Nearly always | 11 (4.8%) | 0 (0.0%) | 11 (3.0%) |
| Relative negatively affects relationship with others |  |  |  |
| Never | 28 (12.3%) | 1 (0.7%) | 29 (7.9%) |
| Rarely | 37 (16.2%) | 25 (18.2%) | 62 (17.0%) |
| Sometimes | 78 (34.2%) | 56 (40.9%) | 134 (36.7%) |
| Frequently | 71 (31.1%) | 53 (38.7%) | 124 (34.0%) |
| Nearly always | 14 (6.1%) | 2 (1.5%) | 16 (4.4%) |
| Strained around relative |  |  |  |
| Never | 21 (9.2%) | 3 (2.2%) | 24 (6.6%) |
| Rarely | 40 (17.5%) | 56 (40.9%) | 96 (26.3%) |
| Sometimes | 92 (40.4%) | 68 (49.6%) | 160 (43.8%) |
| Frequently | 57 (25.0%) | 10 (7.3%) | 67 (18.4%) |
| Nearly always | 18 (7.9%) | 0 (0.0%) | 18 (4.9%) |
| Health suffered because of relative |  |  |  |
| Never | 11 (4.8%) | 3 (2.2%) | 14 (3.8%) |
| Rarely | 15 (6.6%) | 67 (48.9%) | 82 (22.5%) |
| Sometimes | 74 (32.5%) | 60 (43.8%) | 134 (36.7%) |
| Frequently | 67 (29.4%) | 7 (5.1%) | 74 (20.3%) |
| Nearly always | 61 (26.8%) | 0 (0.0%) | 61 (16.7%) |
| Less privacy |  |  |  |
| Never | 34 (14.9%) | 4 (2.9%) | 38 (10.4%) |
| Rarely | 29 (12.7%) | 38 (27.7%) | 67 (18.4%) |
| Sometimes | 61 (26.8%) | 63 (46.0%) | 124 (34.0%) |
| Frequently | 67 (29.4%) | 27 (19.7%) | 94 (25.8%) |
| Nearly always | 37 (16.2%) | 5 (3.6%) | 42 (11.5%) |
| Social life suffered because of relative |  |  |  |
| Never | 3 (1.3%) | 0 (0.0%) | 3 (0.8%) |
| Rarely | 14 (6.1%) | 28 (20.4%) | 42 (11.5%) |
| Sometimes | 52 (22.8%) | 74 (54.0%) | 126 (34.5%) |
| Frequently | 81 (35.5%) | 33 (24.1%) | 114 (31.2%) |
| Nearly always | 78 (34.2%) | 2 (1.5%) | 80 (21.9%) |
| Lost control of life since relative's illness |  |  |  |
| Never | 9 (3.9%) | 2 (1.5%) | 11 (3.0%) |
| Rarely | 20 (8.8%) | 59 (43.1%) | 79 (21.6%) |
| Sometimes | 61 (26.8%) | 60 (43.8%) | 121 (33.2%) |
| Frequently | 77 (33.8%) | 14 (10.2%) | 91 (24.9%) |
| Nearly always | 61 (26.8%) | 2 (1.5%) | 63 (17.3%) |
| Uncertain about what to do about relative |  |  |  |
| Never | 10 (4.4%) | 1 (0.7%) | 11 (3.0%) |
| Rarely | 12 (5.3%) | 62 (45.3%) | 74 (20.3%) |
| Sometimes | 56 (24.6%) | 59 (43.1%) | 115 (31.5%) |
| Frequently | 82 (36.0%) | 15 (10.9%) | 97 (26.6%) |
| Nearly always | 68 (29.8%) | 0 (0.0%) | 68 (18.6%) |
| Should be doing more for relative |  |  |  |
| Never | 6 (2.6%) | 0 (0.0%) | 6 (1.6%) |
| Rarely | 14 (6.1%) | 29 (21.2%) | 43 (11.8%) |
| Sometimes | 46 (20.2%) | 60 (43.8%) | 106 (29.0%) |
| Frequently | 74 (32.5%) | 29 (21.2%) | 103 (28.2%) |
| Nearly always | 88 (38.6%) | 19 (13.9%) | 107 (29.3%) |
| Better job in caring for your relative |  |  |  |
| Never | 10 (4.4%) | 0 (0.0%) | 10 (2.7%) |
| Rarely | 24 (10.5%) | 59 (43.1%) | 83 (22.7%) |
| Sometimes | 63 (27.6%) | 31 (22.6%) | 94 (25.8%) |
| Frequently | 71 (31.1%) | 47 (34.3%) | 118 (32.3%) |
| Nearly always | 60 (26.3%) | 0 (0.0%) | 60 (16.4%) |
| Data are frequency (%). DSRD: Down syndrome regression disorder; DSN: Down syndrome with neurological disorders; and ZCB: Zarit caregiver burden. | | | |
