# Supplementary material for: Caregiver burden and familial impact in Down Syndrome Regression Disorder
Source: Orphanet J Rare Dis. 2025 Mar 14;20:126. doi: 10.1186/s13023-025-03644-0 (PMC11909950; doi:10.1186/s13023-025-03644-0)
Supplement: Supplementary file 3 — Supplementary Material 3 [file 13023_2025_3644_MOESM3_ESM.docx]

| **Table S3**. GDS survey responses by DSRD and DSN caregiver groups. | | | | |
| --- | --- | --- | --- | --- |
|  | DSRD | DSN | Total | |
|  | (n = 228) | (n = 137) | (n = 365) | |
| Felt sad |  |  |  | |
| Never/No | 103 (45.2%) | 108 (78.8%) | 211 (57.8%) | |
| Sometimes | 72 (31.6%) | 24 (17.5%) | 96 (26.3%) | |
| Always/ a lot | 53 (23.2%) | 5 (3.6%) | 58 (15.9%) | |
| Bad mood |  |  |  | |
| Never/No | 128 (56.1%) | 103 (75.2%) | 231 (63.3%) | |
| Sometimes | 73 (32.0%) | 30 (21.9%) | 103 (28.2%) | |
| Always/ a lot | 27 (11.8%) | 4 (2.9%) | 31 (8.5%) | |
| Enjoyed things you've done |  |  |  | |
| Never/No | 121 (53.1%) | 25 (18.2%) | 146 (40.0%) | |
| Sometimes | 92 (40.4%) | 40 (29.2%) | 132 (36.2%) | |
| Always/ a lot | 15 (6.6%) | 72 (52.6%) | 87 (23.8%) | |
| Enjoyed talking to and being with others |  |  |  | |
| Never/No | 126 (55.3%) | 10 (7.3%) | 136 (37.3%) | |
| Sometimes | 73 (32.0%) | 34 (24.8%) | 107 (29.3%) | |
| Always/ a lot | 29 (12.7%) | 93 (67.9%) | 122 (33.4%) | |
| Kept up personal hygiene |  |  |  | |
| Never/No | 90 (39.5%) | 2 (1.5%) | 92 (25.2%) | |
| Sometimes | 29 (12.7%) | 2 (1.5%) | 31 (8.5%) | |
| Always/ a lot | 109 (47.8%) | 133 (97.1%) | 242 (66.3%) | |
| Felt tired during the day |  |  |  | |
| Never/No | 88 (38.6%) | 91 (66.4%) | 179 (49.0%) | |
| Sometimes | 65 (28.5%) | 40 (29.2%) | 105 (28.8%) | |
| Always/ a lot | 75 (32.9%) | 6 (4.4%) | 81 (22.2%) | |
| Cried |  |  |  | |
| Never/No | 138 (60.5%) | 131 (95.6%) | 269 (73.7%) | |
| Sometimes | 71 (31.1%) | 6 (4.4%) | 77 (21.1%) | |
| Always/ a lot | 19 (8.3%) | 0 (0.0%) | 19 (5.2%) | |
| Pay attention/concentrate |  |  |  | |
| Never/No | 116 (50.9%) | 35 (25.5%) | 151 (41.4%) | |
| Sometimes | 67 (29.4%) | 22 (16.1%) | 89 (24.4%) | |
| Always/ a lot | 45 (19.7%) | 80 (58.4%) | 125 (34.2%) | |
| Hard to make decisions |  |  |  | |
| Never/No | 143 (62.7%) | 127 (92.7%) | 270 (74.0%) | |
| Sometimes | 64 (28.1%) | 9 (6.6%) | 73 (20.0%) | |
| Always/ a lot | 21 (9.2%) | 1 (0.7%) | 22 (6.0%) | |
| Hard to sit still |  |  |  | |
| Never/No | 169 (74.1%) | 133 (97.1%) | 302 (82.7%) | |
| Sometimes | 45 (19.7%) | 4 (2.9%) | 49 (13.4%) | |
| Always/ a lot | 14 (6.1%) | 0 (0.0%) | 14 (3.8%) | |
| Eating too little or too much |  |  |  | |
| Never/No | 143 (62.7%) | 125 (91.2%) | 268 (73.4%) | |
| Sometimes | 55 (24.1%) | 10 (7.3%) | 65 (17.8%) | |
| Always/ a lot | 30 (13.2%) | 2 (1.5%) | 32 (8.8%) | |
| Hard to get good sleep |  |  |  | |
| Never/No | 87 (38.2%) | 71 (51.8%) | 158 (43.3%) | |
| Sometimes | 43 (18.9%) | 48 (35.0%) | 91 (24.9%) | |
| Always/ a lot | 98 (43.0%) | 18 (13.1%) | 116 (31.8%) | |
| Life is not worth living |  |  |  | |
| Never/No | 202 (88.6%) | 137 (100.0%) | 339 (92.9%) | |
| Sometimes | 22 (9.6%) | 0 (0.0%) | 22 (6.0%) | |
| Always/ a lot | 4 (1.8%) | 0 (0.0%) | 4 (1.1%) | |
| Felt as if everything is your fault |  |  |  | |
| Never/No | 191 (83.8%) | 135 (98.5%) | 326 (89.3%) | |
| Sometimes | 29 (12.7%) | 2 (1.5%) | 31 (8.5%) | |
| Always/ a lot | 8 (3.5%) | 0 (0.0%) | 8 (2.2%) | |
| Felt that other people were looking/talking/laughing at you |  |  |  | |
| Never/No | 196 (86.0%) | 137 (100.0%) | 333 (91.2%) | |
| Sometimes | 28 (12.3%) | 0 (0.0%) | 28 (7.7%) | |
| Always/ a lot | 4 (1.8%) | 0 (0.0%) | 4 (1.1%) | |
| Very upset if someone disagrees with you or tells you about |  |  |  | |
| Never/No | 168 (73.7%) | 106 (77.4%) | 274 (75.1%) | |
| Sometimes | 49 (21.5%) | 27 (19.7%) | 76 (20.8%) | |
| Always/ a lot | 11 (4.8%) | 4 (2.9%) | 15 (4.1%) | |
| Felt worried |  |  |  | |
| Never/No | 90 (39.5%) | 56 (40.9%) | 146 (40.0%) | |
| Sometimes | 81 (35.5%) | 57 (41.6%) | 138 (37.8%) | |
| Always/ a lot | 57 (25.0%) | 24 (17.5%) | 81 (22.2%) | |
| Thought bad things keep happening to you |  |  |  | |
| Never/No | 163 (71.5%) | 131 (95.6%) | 294 (80.5%) | |
| Sometimes | 39 (17.1%) | 6 (4.4%) | 45 (12.3%) | |
| Always/ a lot | 26 (11.4%) | 0 (0.0%) | 26 (7.1%) | |
| Felt happy when something good happened |  |  |  | |
| Never/No | 95 (41.7%) | 12 (8.8%) | 107 (29.3%) | |
| Sometimes | 67 (29.4%) | 11 (8.0%) | 78 (21.4%) | |
| Always/ a lot | 66 (28.9%) | 114 (83.2%) | 180 (49.3%) | |
| Data are frequency (%). DSRD: Down syndrome regression disorder; DSN: Down syndrome with neurological disorders; and GDS: Glasgow depression scale. | | | |  |
