# Supplementary material for: Caregiver burden and familial impact in Down Syndrome Regression Disorder
Source: Orphanet J Rare Dis. 2025 Mar 14;20:126. doi: 10.1186/s13023-025-03644-0 (PMC11909950; doi:10.1186/s13023-025-03644-0)
Supplement: Supplementary file 4 — Supplementary Material 4 [file 13023_2025_3644_MOESM4_ESM.docx]

| **Table S4**. CDS responses on primary positive support across DSRD and DSN caregiver groups. | | | |
| --- | --- | --- | --- |
|  | DSRD | DSN | Total |
|  | (n = 228) | (n = 137) | (n = 365) |
| *Before my child’s/relative’s diagnosis:* |  |  |  |
| Primary support = friends |  |  |  |
| No | 19 (8.3%) | 11 (8.0%) | 30 (8.2%) |
| Yes | 209 (91.7%) | 126 (92.0%) | 335 (91.8%) |
| Primary support = family |  |  |  |
| No | 23 (10.1%) | 11 (8.0%) | 34 (9.3%) |
| Yes | 205 (89.9%) | 126 (92.0%) | 331 (90.7%) |
| Primary support = siblings |  |  |  |
| No | 88 (38.6%) | 57 (41.6%) | 145 (39.7%) |
| Yes | 140 (61.4%) | 80 (58.4%) | 220 (60.3%) |
| Primary support = church |  |  |  |
| No | 131 (57.5%) | 79 (57.7%) | 210 (57.5%) |
| Yes | 97 (42.5%) | 58 (42.3%) | 155 (42.5%) |
| Primary support = medical teams |  |  |  |
| No | 153 (67.1%) | 91 (66.4%) | 244 (66.8%) |
| Yes | 75 (32.9%) | 46 (33.6%) | 121 (33.2%) |
| Primary support = local community |  |  |  |
| No | 146 (64.0%) | 92 (67.2%) | 238 (65.2%) |
| Yes | 82 (36.0%) | 45 (32.8%) | 127 (34.8%) |
| Primary support = online community |  |  |  |
| No | 161 (70.6%) | 94 (68.6%) | 255 (69.9%) |
| Yes | 67 (29.4%) | 43 (31.4%) | 110 (30.1%) |
| Primary support = local DS organizations |  |  |  |
| No | 140 (61.4%) | 80 (58.4%) | 220 (60.3%) |
| Yes | 88 (38.6%) | 57 (41.6%) | 145 (39.7%) |
| Primary support = national DS organizations |  |  |  |
| No | 186 (81.6%) | 116 (84.7%) | 302 (82.7%) |
| Yes | 42 (18.4%) | 21 (15.3%) | 63 (17.3%) |
| Primary support = other |  |  |  |
| No | 213 (93.4%) | 131 (95.6%) | 344 (94.2%) |
| Yes | 15 (6.6%) | 6 (4.4%) | 21 (5.8%) |
| *After my child’s/relative’s diagnosis:* |  |  |  |
| Primary support = friends |  |  |  |
| No | 94 (41.2%) | 30 (21.9%) | 124 (34.0%) |
| Yes | 134 (58.8%) | 107 (78.1%) | 241 (66.0%) |
| Primary support = family |  |  |  |
| No | 60 (26.3%) | 11 (8.0%) | 71 (19.5%) |
| Yes | 168 (73.7%) | 126 (92.0%) | 294 (80.5%) |
| Primary support = siblings |  |  |  |
| No | 134 (58.8%) | 61 (44.5%) | 195 (53.4%) |
| Yes | 94 (41.2%) | 76 (55.5%) | 170 (46.6%) |
| Primary support = church |  |  |  |
| No | 189 (82.9%) | 87 (63.5%) | 276 (75.6%) |
| Yes | 39 (17.1%) | 50 (36.5%) | 89 (24.4%) |
| Primary support = medical teams |  |  |  |
| No | 143 (62.7%) | 84 (61.3%) | 227 (62.2%) |
| Yes | 85 (37.3%) | 53 (38.7%) | 138 (37.8%) |
| Primary support = local community |  |  |  |
| No | 210 (92.1%) | 98 (71.5%) | 308 (84.4%) |
| Yes | 18 (7.9%) | 39 (28.5%) | 57 (15.6%) |
| Primary support = online community |  |  |  |
| No | 104 (45.6%) | 100 (73.0%) | 204 (55.9%) |
| Yes | 124 (54.4%) | 37 (27.0%) | 161 (44.1%) |
| Primary support = local DS organizations |  |  |  |
| No | 198 (86.8%) | 96 (70.1%) | 294 (80.5%) |
| Yes | 30 (13.2%) | 41 (29.9%) | 71 (19.5%) |
| Primary support = national DS organizations |  |  |  |
| No | 214 (93.9%) | 121 (88.3%) | 335 (91.8%) |
| Yes | 14 (6.1%) | 16 (11.7%) | 30 (8.2%) |
| Primary support = other |  |  |  |
| No | 208 (91.2%) | 133 (97.1%) | 341 (93.4%) |
| Yes | 20 (8.8%) | 4 (2.9%) | 24 (6.6%) |
| Data are frequency (%). DSRD: Down syndrome regression disorder; DSN: Down syndrome with neurological disorders; and CDS: Caregiver distress survey. | | | |
