# Supplementary material for: Caregiver burden and familial impact in Down Syndrome Regression Disorder
Source: Orphanet J Rare Dis. 2025 Mar 14;20:126. doi: 10.1186/s13023-025-03644-0 (PMC11909950; doi:10.1186/s13023-025-03644-0)
Supplement: Supplementary file 5 — Supplementary Material 5 [file 13023_2025_3644_MOESM5_ESM.docx]

| **Table S5**. Estimated regression coefficients with 95% confidence intervals (CIs) for PedsQL, ZCB, and GDS scores, and odds ratios (ORs) with 95% CI for GDS (≥ 13 vs. < 13), across DSRD and DSN caregiver groups, stratified by potential CDS factors. | | | | | | |
| --- | --- | --- | --- | --- | --- | --- |
|  | | DSRD vs. DSN | | | | |
|  | | Coef. | SE | [95% CI] | | *p* |
| *PedsQL: Total score* | |  |  |  |  |  |
| Age | | -0.13 | 0.21 | [-0.54, | 0.27] | 0.522 |
| Sex at birth: Male vs. Female | | -0.09 | 2.41 | [-4.84, | 4.65] | 0.969 |
| Ethnicity: Hispanic vs. Non-Hispanic | | -2.19 | 2.65 | [-7.40, | 3.02] | 0.409 |
| Duration of symptoms | | -0.11 | 0.48 | [-1.05, | 0.83] | 0.817 |
| Increased financial burden: Yes vs. No^*^ | | -5.01 | 2.41 | [-9.74, | -0.27] | **0.038** |
|  | Yes | -29.57 | 1.60 | [-32.71, | -26.43] | **<0.001** |
|  | No | -24.56 | 1.80 | [ -28.10, | -21.02] | **<0.001** |
| Change housing: Yes vs. No | | -0.08 | 3.28 | [-6.53, | 6.37] | 0.981 |
| Disrupted sleep: Yes vs. No^*^ | | -6.30 | 2.83 | [-11.87, | -0.73] | **0.027** |
|  | Yes | -29.60 | 2.13 | [-33.79, | -25.41] | **<0.001** |
|  | No | -23.30 | 1.87 | [ -26.97, | -19.63] | **<0.001** |
| Social networks impacted: Negative change vs. Positive or no change^*^ | | -6.66 | 2.50 | [-11.58, | -1.73] | **0.008** |
|  | Negative change | -29.63 | 1.79 | [-33.15, | -26.12] | **<0.001** |
|  | Positive or no change | -22.98 | 1.75 | [-26.42, | -19.53] | **<0.001** |
| Worsened mental health: Yes vs. No^*^ | | -10.50 | 2.80 | [-16.01, | -4.99] | **<0.001** |
|  | Yes | -30.07 | 2.13 | [-34.27, | -25.88] | **<0.001** |
|  | No | -19.57 | 1.82 | [-23.14, | -16.00] | **<0.001** |
| *PedsQL: The parent HRQL summary score* | |  |  |  |  |  |
| Age | | -0.15 | 0.24 | [-0.62, | 0.31] | 0.512 |
| Sex at birth: Male vs. Female | | 1.02 | 2.74 | [-4.37, | 6.41] | 0.710 |
| Ethnicity: Hispanic vs. Non-Hispanic | | -0.28 | 3.01 | [-6.20, | 5.64] | 0.926 |
| Duration of symptoms | | -0.21 | 0.54 | [-1.28, | 0.85] | 0.695 |
| Increased financial burden: Yes vs. No | | -4.24 | 2.75 | [-9.64, | 1.17] | 0.124 |
| Change housing: Yes vs. No | | 0.77 | 3.74 | [-6.58, | 8.13] | 0.836 |
| Disrupted sleep: Yes vs. No^*^ | | -6.88 | 3.22 | [-13.20, | -0.56] | **0.033** |
|  | Yes | -30.07 | 2.13 | [-34.27, | -25.88] | **<0.001** |
|  | No | -19.57 | 1.82 | [-23.14, | -16.00] | **<0.001** |
| Social networks impacted: Negative change vs. Positive or no change^*^ | | -7.34 | 2.86 | [-12.95, | -1.72] | **0.011** |
|  | Negative change | -33.62 | 2.04 | [-37.63, | -29.61] | **<0.001** |
|  | Positive or no change | -26.28 | 2.00 | [-30.22, | -22.35] | **<0.001** |
| Worsened mental health: Yes vs. No^*^ | | -11.70 | 3.18 | [-17.95, | -5.45] | **<0.001** |
|  | Yes | -33.99 | 2.42 | [-38.75, | -29.23] | **<0.001** |
|  | No | -22.29 | 2.06 | [-26.34, | -18.24] | **<0.001** |
| *PedsQL: The family functioning summary score* | |  |  |  |  |  |
| Age | | 0.06 | 0.29 | [-0.51, | 0.63] | 0.838 |
| Sex at birth: Male vs. Female | | -1.12 | 3.36 | [-7.74, | 5.50] | 0.739 |
| Ethnicity: Hispanic vs. Non-Hispanic | | -4.60 | 3.69 | [-11.85, | 2.66] | 0.213 |
| Duration of symptoms | | 0.02 | 0.66 | [-1.29, | 1.33] | 0.977 |
| Increased financial burden: Yes vs. No^*^ | | -9.03 | 3.34 | [-15.60, | -2.46] | **0.007** |
|  | Yes | -22.27 | 2.22 | [-26.63, | -17.91] | **<0.001** |
|  | No | -13.24 | 2.50 | [ -18.16, | -8.33] | **<0.001** |
| Change housing: Yes vs. No | | -4.49 | 4.55 | [-13.44, | 4.46] | 0.325 |
| Disrupted sleep: Yes vs. No | | -6.77 | 3.98 | [-14.59, | 1.05] | *0.089* |
| Social networks impacted: Negative change vs. Positive or no change^*^ | | -8.15 | 3.50 | [-15.03, | -1.27] | **0.020** |
|  | Negative change | -20.90 | 2.50 | [-25.81, | -15.99] |  |
|  | Positive or no change | -12.75 | 2.45 | [-17.57, | -7.93] |  |
| Worsened mental health: Yes vs. No^*^ | | -11.54 | 4.02 | [-19.44, | -3.64] | **0.004** |
|  | Yes | -22.42 | 3.06 | [ -28.44, | -16.41] | **<0.001** |
|  | No | -10.88 | 2.61 | [-16.00, | -5.76] | **<0.001** |
| *Zarit caregiver burden total score* | |  |  |  |  |  |
| Age | | -0.00 | 0.12 | [-0.24, | 0.23] | 0.978 |
| Sex at birth: Male vs. Female | | 0.86 | 1.40 | [-1.90, | 3.62] | 0.541 |
| Ethnicity: Hispanic vs. Non-Hispanic | | 0.90 | 1.54 | [-2.14, | 3.93] | 0.562 |
| Duration of symptoms | | 0.13 | 0.28 | [-0.41, | 0.68] | 0.632 |
| Increased financial burden: Yes vs. No^*^ | | 4.90 | 1.39 | [2.18, | 7.63] | **<0.001** |
|  | Yes | 10.07 | 0.92 | [ 8.26, | 11.87] | **<0.001** |
|  | No | 5.16 | 1.04 | [ 3.12, | 7.20] | **<0.001** |
| Change housing: Yes vs. No | | 3.17 | 1.90 | [-0.56, | 6.90] | *0.096* |
| Disrupted sleep: Yes vs. No | | 3.07 | 1.64 | [-0.16, | 6.30] | *0.062* |
| Social networks impacted: Negative change vs. Positive or no change | | 3.04 | 1.49 | [0.12, | 5.96] | **0.041** |
|  | Negative change | 9.24 | 1.06 | [7.16, | 11.33] | **<0.001** |
|  | Positive or no change | 6.20 | 1.04 | [4.15, | 8.24] | **<0.001** |
| Worsened mental health: Yes vs. No^*^ | | 6.59 | 1.62 | [3.40, | 9.78] | **<0.001** |
|  | Yes | 10.02 | 1.24 | [7.59, | 12.44] | **<0.001** |
|  | No | 3.43 | 1.05 | [1.36, | 5.50] | **0.001** |
| *Glasgow depression score*^†^ | |  |  |  |  |  |
| Age | | 0.00 | 0.11 | [-0.21, | 0.21] | 0.999 |
| Sex at birth: Male vs. Female | | -2.00 | 1.65 | [-5.24, | 1.24] | 0.225 |
| Ethnicity: Hispanic vs. Non-Hispanic | | 0.00 | 1.73 | [-3.39, | 3.39] | 0.999 |
| Duration of symptoms | | -0.12 | 0.30 | [-0.72, | 0.47] | 0.680 |
| Increased financial burden: Yes vs. No^*^ | | 5.00 | 1.61 | [1.84, | 8.16] | **0.002** |
|  | Yes | 4.00 | 0.64 | [2.74, | 5.26] | **<0.001** |
|  | No | -1.00 | 1.47 | [-3.88, | 1.88] | 0.497 |
| Change housing: Yes vs. No | | 2.00 | 1.97 | [-1.88, | 5.88] | 0.311 |
| Disrupted sleep: Yes vs. No | | 3.00 | 2.04 | [-1.02, | 7.02] | 0.143 |
| Social networks impacted: Negative change vs. Positive or no change | | 1.00 | 1.93 | [-2.80, | 4.80] | 0.605 |
| Worsened mental health: Yes vs. No | | 5.00 | 3.45 | [-1.79, | 11.79] | 0.149 |
|  | | DSRD vs. DSN | | | | |
| *Glasgow depression score (≥ 13 vs. < 13)* | | OR | SE | [95% CI] | | *p* |
| Age | | 0.97 | 0.04 | [0.89, | 1.06] | 0.514 |
| Sex at birth: Male vs. Female^*^ | | 0.16 | 0.10 | [0.05, | 0.53] | **0.002** |
|  | Male | 0.21 | 0.07 | [0.07, | 0.35] | **0.002** |
|  | Female | 0.49 | 0.06 | [0.38, | 0.61] | **<0.001** |
| Ethnicity: Hispanic vs. Non-Hispanic | | 1.85 | 1.09 | [0.58, | 5.86] | 0.297 |
| Duration of symptoms | | 0.93 | 0.10 | [0.76, | 1.14] | 0.485 |
| Increased financial burden: Yes vs. No | | 2.11 | 1.09 | [0.77, | 5.80] | 0.149 |
| Change housing: Yes vs. No | | 0.58 | 0.37 | [0.17, | 2.05] | 0.401 |
| Disrupted sleep: Yes vs. No | | 3.20 | 2.14 | [0.87, | 11.85] | 0.081 |
| Social networks impacted: Negative change vs. Positive or no change | | 0.56 | 0.30 | [0.20, | 1.58] | 0.274 |
| Worsened mental health: Yes vs. No | | 0.84 | 0.49 | [0.27, | 2.61] | 0.768 |
| ^*^*p* < 0.05 (in bold font) and *p* < 0.1 (in italic font). ^†^Quantile regression model was performed for GDS.  DSRD: Down syndrome regression disorder; DSN: Down syndrome with neurological disorders; PedsQL: Pediatric quality of life – Family impact module; HRQL: Health-related quality of life; ZCB: Zarit caregiver burden; GDS: Glasgow depression scale; and CDS: Caregiver distress survey. | | | | | | |
