# Supplementary material for: Caregiver burden and familial impact in Down Syndrome Regression Disorder
Source: Orphanet J Rare Dis. 2025 Mar 14;20:126. doi: 10.1186/s13023-025-03644-0 (PMC11909950; doi:10.1186/s13023-025-03644-0)
Supplement: Supplementary file 6 — Supplementary Material 6 [file 13023_2025_3644_MOESM6_ESM.docx]

| (a) | (b) | (c) |
| --- | --- | --- |
|  |  |  |
| (d) | (e) | (f) |
|  |  |  |
| (g) | (h) | (i) |
|  |  |  |
| (j) | (k) | (l) |
|  |  |  |
| (m) | (n) | (o) |
|  |  |  |
| (p) | (q) | (r) |
|  |  |  |
| (s) | (t) | (v) |
|  |  |  |
| (w) | (x) | (y) |
|  |  |  |

**Figure S1.** Significant associations between PedsQL total score, parent HRQL summary score, family functioning summary score, ZCB score, GDS, and PedsQL subdomains with key CDS factors—including worsened mental health, disrupted sleep, increased financial burden, and impacted social networks—among DSRD and DSN caregiver groups. DSRD: Down syndrome regression disorder; DSN: Down syndrome with neurological disorders; PedsQL: Pediatric quality of life – Family impact module; HRQL: Health-related quality of life; ZCB: Zarit caregiver burden; GDS: Glasgow depression scale; and CDS: Caregiver distress survey.
